# Comparison of Control of *Clostridium difficile* Infection in Six English Hospitals Using Whole-Genome Sequencing

**DOI:** 10.1093/cid/cix338

**Published:** 2017-05-29

**Authors:** David W. Eyre, Warren N. Fawley, Anu Rajgopal, Christopher Settle, Kalani Mortimer, Simon D. Goldenberg, Susan Dawson, Derrick W. Crook, Tim E. A. Peto, A. Sarah Walker, Mark H. Wilcox

**Affiliations:** 1 Nuffield Department of Medicine, University of Oxford,; 2 Department of Microbiology, Leeds Teaching Hospitals NHS Trust,; 3 Calderdale and Huddersfield NHS Foundation Trust,; 4 City Hospitals Sunderland NHS Foundation Trust,; 5 St. Helens and Knowsley Teaching Hospitals NHS Trust, Merseyside,; 6 Guy’s & St Thomas’ NHS Foundation Trust, London, and; 7 Great Western Hospitals NHS Foundation Trust, Swindon, United Kingdom

**Keywords:** infection control, *Clostridium difficile*, whole-genome sequencing, transmission, surveillance.

## Abstract

**Background.:**

Variation in *Clostridium difficile* infection (CDI) rates between healthcare institutions suggests overall incidence could be reduced if the lowest rates could be achieved more widely.

**Methods.:**

We used whole-genome sequencing (WGS) of consecutive *C. difficile* isolates from 6 English hospitals over 1 year (2013–14) to compare infection control performance. Fecal samples with a positive initial screen for *C. difficile* were sequenced. Within each hospital, we estimated the proportion of cases plausibly acquired from previous cases.

**Results.:**

Overall, 851/971 (87.6%) sequenced samples contained toxin genes, and 451 (46.4%) were fecal-toxin-positive. Of 652 potentially toxigenic isolates >90-days after the study started, 128 (20%, 95% confidence interval [CI] 17–23%) were genetically linked (within ≤2 single nucleotide polymorphisms) to a prior patient’s isolate from the previous 90 days. Hospital 2 had the fewest linked isolates, 7/105 (7%, 3–13%), hospital 1, 9/70 (13%, 6–23%), and hospitals 3–6 had similar proportions of linked isolates (22–26%) (P ≤ .002 comparing hospital-2 vs 3–6). Results were similar adjusting for locally circulating ribotypes. Adjusting for hospital, ribotype-027 had the highest proportion of linked isolates (57%, 95% CI 29–81%). Fecal-toxin-positive and toxin-negative patients were similarly likely to be a potential transmission donor, OR = 1.01 (0.68–1.49). There was no association between the estimated proportion of linked cases and testing rates.

**Conclusions.:**

WGS can be used as a novel surveillance tool to identify varying rates of *C. difficile* transmission between institutions and therefore to allow targeted efforts to reduce CDI incidence.

## Summary

Infection control effectiveness at 6 English hospitals was compared using whole-genome sequencing of *C. difficile* isolates. Proportions of isolates sufficiently genetically related to suggest transmission from a previous isolate ranged from 7% at the best institution to 22–26% in most others.

Preventing *Clostridium difficile* infection (CDI) is a priority for infection control teams, as it remains a major healthcare-associated infection; although the incidence of healthcare-associated CDI in the United Kingdom has fallen to 1.5 per 10000 inpatient bed-days [1], rates across Europe range from 0.7 to 28.7/10000 bed-days [2], and there were an estimated 293000 healthcare-associated cases in the United States in 2011 [3].

Variation in CDI incidence across countries and between healthcare institutions [4] suggests overall incidence could be reduced if the lowest rates could be achieved more widely. Surveillance programs [5] and penalties for healthcare institutions [6] have been implemented to promote reductions. However, robustly identifying the best performing institutions is challenging. Variations in true incidence can arise from differences in patient risk factors or locally circulating strains. However, testing strategy also influences reported incidence; reported CDI incidence is associated with testing rates [2]. With low testing rates, CDI ascertainment is likely to be suboptimal. Conversely, high testing rates may lead to overdiagnosis, for example, from testing *C. difficile* colonized patients, who do not have CDI but may have diarrhea of another cause. The lack of a universally accepted objective CDI case definition means that robust comparisons of infection rates between institutions should ideally also consider independent measures of which patients are being tested to assess the comparability of differing testing strategies [7].

Additionally, assessing potential sources of healthcare- attributed CDI cases [8] is complex, requiring differentiation between lapses in infection control around symptomatic cases or more generally, deviation from optimal antimicrobial stewardship, and external factors, for example, the food chain. Healthcare exposure increases the risk of *C. difficile* acquisition; both CDI and colonization increase during hospital stay [9]. However, despite this strong association, studies using whole-genome sequencing (WGS) [10–12] and other genotyping schemes [13–15] have shown that, in endemic settings with standard infection control, only the minority of infections are likely to have been acquired from other hospitalized CDI cases. However, the extent to which this proportion of linked cases varies between hospitals is unknown. Furthermore, such potential variance in linkage rates could identify a potentially preventable group of CDIs.

We investigated variation in the proportion of linked cases using WGS of consecutive *C. difficile* isolates from 6 hospitals in England and explored whether this could be used to assess their infection control effectiveness, by assessing the proportion of cases plausibly acquired from (linked to) previous cases.

## METHODS

### Samples and Settings

Hospitals in England are recommended to store frozen aliquots of *C. difficile–*positive fecal samples for 12 months [16]. Stored consecutive hospital and community diarrheal samples submitted for routine *C. difficile* testing at 6 hospital laboratories were studied, including a tertiary referral center and teaching hospital, and 5 district general hospitals serving a mix of urban and rural populations (see Supplement). Samples were obtained for a one-year period at each hospital between January 2013 and October 2014. Results were anonymized by assigning a computer-generated random identifier, hospital 1 to hospital 6.

Each hospital used the United Kingdom-recommended 2-stage *C. difficile* testing algorithm [17]. Hospital 1 used toxin gene polymerase chain reaction (PCR) as a screening test, hospital 2 both glutamate dehydrogenase (GDH) enzyme immunoassay (EIA) and toxin gene PCR as a combined screening test, and hospitals 3–6 a GDH screen. Screen-positive samples underwent confirmatory fecal-toxin EIA testing. Screen-positive, fecal-toxin-positive patients were regarded as having CDI. Toxin gene PCR was also performed as a third-line test on all GDH-positive samples at hospitals 3 and 6, and on samples from inpatients at hospital 5. PCR-positive, fecal-toxin-negative patients, with a clinical syndrome in keeping with CDI, were regarded as potential cases for treatment and infection control purposes.

All screen-positive fecal samples were sent to Leeds General Infirmary microbiology laboratory, United Kingdom (except hospital 2, which submitted isolates and excluded toxin EIA-negative/PCR-negative samples), where they underwent selective culture for *C. difficile* [18] and capillary electrophoresis ribotyping [19]. Individual patient consent for use of anonymized bacterial isolates was not required.

### Sequencing

DNA was extracted from subculture of a single colony from each culture-positive sample and sequenced using Illumina HiSeq2500. Sequence data were processed as previously (see Supplement) [10, 20], mapping sequenced reads to the *C. difficile* 630 reference genome [21]. Sequences were compared using single-nucleotide polymorphisms (SNPs) between sequences obtained from maximum-likelihood phylogenies [22], corrected for recombination [23]. Potentially toxigenic strains were identified as those containing toxin genes using BLAST searches of *de novo* [24] assemblies.

### Analysis

For each sample, only the hospital, collection date, and fecal-toxin EIA result were known; no further epidemiological data were available. Within each hospital, sequences were compared with all sequences from samples obtained in the prior 90 days. Samples from the community and hospital were included to increase the chance of identifying transmission events occurring in hospital but leading to CDI onset after discharge. From previous estimates of *C. difficile* evolution and within-host diversity [10, 25, 26], ≤2 SNPs are expected between isolates linked by transmission within 90 days. Therefore, where ≥1 prior sequences within ≤2 SNPs were identified, a case was considered to have been potentially acquired from another case. A 90 day threshold for linking cases was chosen assuming that cases were rapidly treated and infectiousness declined, and that subsequent cases related by direct transmission occurred within incubation periods implied by surveillance definitions [8] and previous studies [13]. As the sources of cases occurring at the start of the study may themselves have been sampled before the study started, the proportion of cases linked to a prior case was only calculated for cases occurring after the first 90 days, with cases in the first 90 days included only as potential sources for subsequent cases.

Two differing case definitions were considered. Initially, all patients with culture-positive potentially toxigenic *C. difficile* were considered “cases” to capture possible transmission events involving potentially toxigenic *C. difficile* irrespective of fecal-toxin status. The analysis was then repeated restricted only to fecal-toxin-positive CDI cases. For comparisons with previously published data, the same definition and analysis approach was applied to fecal-toxin-positive CDI cases occurring within 90 days in Oxford (September 2007 to December 2010, split by calendar year) [10] and Leeds (August 2010 to April 2012) [11].

### Risk Factor Analysis

Univariate logistic regression was used to determine whether a case’s toxin status affected the risk of it being genetically related to a prior case, that is, potentially acquired from another case. Similarly, logistic regression was used to determine whether a case’s fecal-toxin status affected the risk of it being genetically linked to a subsequent case, that is, to assess the relative infectiousness of fecal-toxin-positive and toxin-negative patients.

To assess whether the locally circulating strain mix affected transmission estimates, hospital-specific estimates were adjusted for ribotype using multivariate logistic regression (see Supplement).

### Simulations

To estimate the impact of missing data (as not all sampled cases were sequenced at some hospitals), we simulated transmission at a theoretical hospital. We subsampled simulated cases and calculated the change in the percentage of cases linked to a prior case as the proportion of missing samples increases (details in Supplement).

## RESULTS

Consecutive samples sent for *C. difficile* testing at 6 hospitals were studied for 12 months (Table 1). In total, 1052/1098 (96%) of GDH/toxin-PCR screen-positive samples were available: 95/98 (97%) at hospital 1, 144/178 (81%) at hospital 2, 118/127 (93%) at hospital 5 and otherwise 100%. 974/1052 (93%) available samples were confirmed as *C. difficile* on culture. For the 5 hospitals with available testing data, 887/21539 (4.1%) of samples submitted for testing were culture-positive (Table 1); 971/974 (99.7%) culture-positive samples were successfully sequenced. Of sequenced culture-positive samples, 451/971 (46.4%) were EIA fecal-toxin-positive, 35–71% by hospital. By contrast, 851/971 (87.6%) were potentially toxigenic, that is, had toxin genes detected via sequence data. Hence, 400/851 (47.0%) samples containing potentially toxigenic *C. difficile* did not have fecal-toxin detected. In the 971 sequenced isolates, the most common ribotypes identified were 014, 015, 005, 002, 020, and 078 (Table 2). Ribotype-027(NAP1/ST-1) only accounted for 16 (2%) cases.

**Table 1.** Hospitals and Samples

Hospital	Dates	Bed- Days	Specimens Tested	Tests Per 10000 Bed- Days	GDH/PCR Screen- Positive, n (% of Tested)	Screen-Positive Samples Stored, n (% of Screen- Positive)	*Clostridium difficile* on Culture, n (% of Stored Screen Positive) [% of Tested]	Successfully Sequenced, n (% of Culture- Positive)	Fecal EIA Toxin- Positive (all Samples) n (% of Screen- Positive)	Fecal EIA Toxin- Positive (Sequenced Samples) n (% of Sequenced)	Toxin Gene on WGS, n (% of Sequenced)	Culture- Positive *C. difficile* Per 10000 Bed-Days	Potentially Toxigenic *C. difficile* Per 10000 Bed-Days	Fecal-Toxin- Positive *C. difficile* Per 10000 Bed-Days
1	Jun 2013– Jul 2014	—	—	—	98	95 (97)	87 (92)	87 (100)	65 (66)	62 (71)	87 (100)	—	—	—
2	Jan 2013– Dec 2013	349338	3439	98	178 (5.2)	144 (81)	143 (99) [4.2]	143 (100)	64 (36)	64 (45)	136 (95)	5.1	4.8	1.8
3	Nov 2013– Oct 2014	216769	5187	239	223 (4.3)	223 (100)	208 (93) [4.0]	206 (99)	123 (55)	115 (56)	175 (85)	9.6	8.2	5.7
4	Sep 2013– Aug 2014	439426	5818	132	288 (5.0)	288 (100)	245 (85) [4.2]	245 (100)	97 (34)	93 (38)	183 (75)	5.6	4.2	2.2
5	Aug 2013– Jul 2014	170733	2949	173	127 (4.3)	118 (93)	113 (96) [3.8]	112 (99)	58 (46)	54 (48)	108 (96)	7.1	6.9	3.4
6	Sep 2013– Aug 2014	252351	4146	164	184 (4.4)	184 (100)	178 (97) [4.3]	178 (100)	63 (34)	63 (35)	162 (91)	7.1	6.4	2.5

Bed-day and specimens tested data were not available for hospital 1. All hospitals used a GDH EIA assay as the initial screening test, except hospital 1, which used a toxin PCR, and hospital 2, which used both GDH EIA and toxin gene PCR as a combined screening test. Culture-positive and potentially toxigenic *C. difficile* rates per 10000 bed-days are shown corrected for the proportion of samples stored, and additionally for potentially toxigenic *C. difficile* the proportion of samples sequenced.

Abbreviations: EIA, enzyme immunoassay; GDH, glutamate dehydrogenase; PCR, polymerase chain reaction; WGS, whole-genome sequencing.

**Table 2.** Ribotype Distribution by Hospital and Proportion of Cases Genetically Linked to a Previous Case by Ribotype

	All		Hospital 1		Hospital 2		Hospital 3		Hospital 4		Hospital 5		Hospital 6		Linked to a Previous Case Within ≤2 SNPs and ≤90 Days		
Ribotype	n	%	n	%	N	%	n	%	n	%	n	%	N	%	n, >90 Days Into Study	n, Linked	% Linked
014	98	10	12	14	18	13	19	9	26	11	10	9	13	7	75	15	20
015	89	9	13	15	14	10	13	6	16	7	18	16	15	8	67	8	12
005	80	8	6	7	9	6	16	8	25	10	10	9	14	8	61	7	11
002	77	8	8	9	11	8	15	7	14	6	6	5	23	13	54	14	26
020	62	6	2	2	11	8	21	10	15	6	5	4	8	4	46	9	20
078	53	5	6	7	9	6	11	5	8	3	6	5	13	7	41	8	20
039	45	5	0	0	5	3	8	4	26	11	1	1	5	3	35	11	31
023	35	4	8	9	4	3	7	3	8	3	3	3	5	3	28	6	21
001	29	3	4	5	1	1	13	6	5	2	3	3	3	2	24	8	33
012	27	3	1	1	5	3	3	1	9	4	7	6	2	1	22	11	50
026	26	3	1	1	1	1	11	5	5	2	1	1	7	4	19	1	5
010	23	2	0	0	0	0	6	3	16	7	0	0	1	1	18	2	11
011	19	2	2	2	10	7	2	1	1	0	2	2	2	1	16	2	13
087	19	2	0	0	0	0	4	2	2	1	7	6	6	3	18	9	50
050	16	2	4	5	1	1	3	1	4	2	2	2	2	1	13	2	15
013	16	2	2	2	4	3	3	1	0	0	6	5	1	1	12	1	8
027	16	2	4	5	2	1	4	2	1	0	0	0	5	3	12	7	58
003	15	2	1	1	2	1	1	0	5	2	0	0	6	3	14	3	21
017	15	2	1	1	0	0	2	1	6	2	1	1	5	3	10	4	40
Other	211	22	12	14%	36	25	44	21	53	22	24	21	42	24	163	19	12
Total	971	100%	87	100%	143	100%	206	100	245	100	112	100	178	100	748	147	20

Abbreviation: SNP, single-nucleotide polymorphism.

### Relatedness to Prior Cases

**Figure 1.**
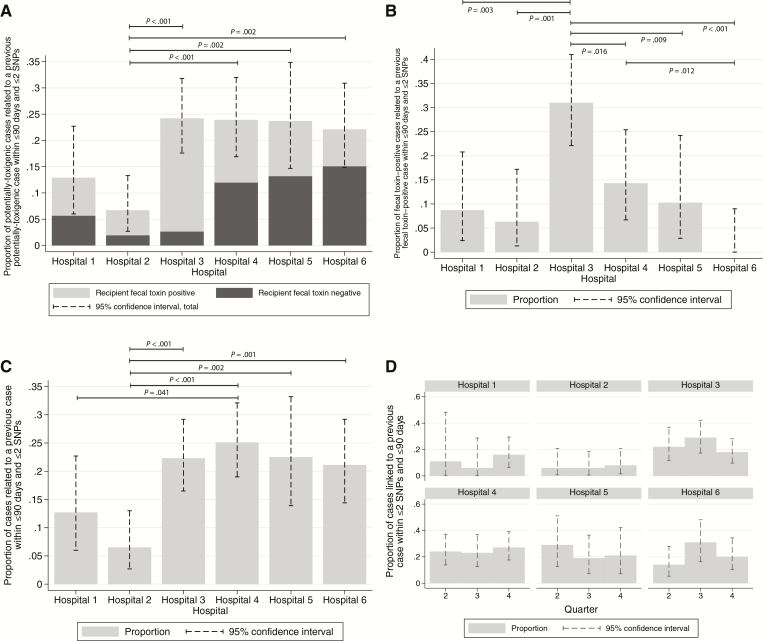
Proportion of cases linked to a previous case by hospital. *A*, Proportion of potentially toxigenic cases linked to a previous potentially toxigenic case, by hospital and recipient fecal-toxin status. *B*, Proportion of fecal-toxin-positive cases linked to a previous fecal-toxin-positive case. *C*, Proportion of all *Clostridium difficile* (potentially toxigenic or nontoxigenic) linked to previous *C. difficile* case. *D*, Proportion of potentially toxigenic cases linked to a previous potentially toxigenic case, by hospital and 90 day period (comparing subsequent quarters to quarter 2 by hospital, hospital 6, quarter 3, *P* = .08, otherwise *P* > .36). Abbreviation: SNP, single-nucleotide polymorphism.

The proportion of cases plausibly linked to a prior case by recent transmission varied by hospital. Of 851 sequenced potentially toxigenic cases, all were considered as potential sources of infection, but only the 652 obtained after the first 90 days of sampling at each hospital were assessed for linkage to a previous case. Across the 6 hospitals, 128/652 (20%, 95% confidence interval [CI] 17–23%) potentially toxigenic cases were genetically linked to a prior case from the previous 90 days. Hospital 2 had the fewest cases linked to a prior case, 7/105 (7%, 3–13%), hospital 1 had an intermediate number, 9/70 (13%, 6–23%), and hospitals 3–6 had similar numbers of linked cases, 37/153 (24%, 18–32%), 32/134 (24%, 17–32%), 18/76 (24%, 15–35%), and 25/113 (22%, 15–31%), respectively. Hospital 2 had significantly fewer linked cases than hospitals 3–6 (*P* ≤ .002), with weaker evidence for lower rates in hospital 1 than hospitals 3, 4, and 5 (*P* = .05, .07, .09, respectively). Overall, 48/128 (38%) of potential transmission recipients were fecal-toxin-negative (11–68% across hospitals, Figure 1A). Fecal-toxin detection in a recipient was associated with increased odds of having a potential transmission donor, odds ratio 1.67 (95% CI 1.12–2.48, *P* = .01).

In total, 59/128 (46%) putative transmission recipients were only linked to ≥1 fecal-toxin-positive potential donors, 50 (39%) to only fecal-toxin-negative donors, and 19 (15%) to both toxin-positive and toxin-negative donors. Considering the 667 cases occurring in the first 270 days at each hospital, that is, the cases with an opportunity to transmit to a sampled case within the next 90 days, 120 (18%) were potential donors. Fecal-toxin-positive and -negative cases were similarly infectious: the odds ratio for a fecal-toxin- positive case, compared to a fecal-toxin-negative case, being a potential transmission donor was 1.01 (95% CI 0.68–1.49, *P* = .97).

When only considering transmission to and from fecal- toxin-positive cases, fewer cases were genetically linked to a previous case within 90 days, 51/335 (15%, 95% CI 12–20%). We observed a different “ranking” of hospitals compared with the above analysis of linkage rates based on potentially toxigenic isolate-positive patients: hospital 3 had the greatest proportion of fecal-toxin-positive cases genetically related to a prior fecal-toxin-positive case, 31% (22–41%), and hospital 6 the lowest, 0% (0–9%) (Figure 1B).

Results were similar to those for all potentially toxigenic *C. difficile* (Figure 1A) if all *C. difficile* sequences, nontoxigenic as well as potentially toxigenic, were considered (Figure 1C). Considering only nontoxigenic isolates, very similarly to potentially toxigenic isolates, 19/96 (20%, 95% CI 12–29%) were genetically linked to a prior patient isolate from the previous 90 days.

**Figure 2.**
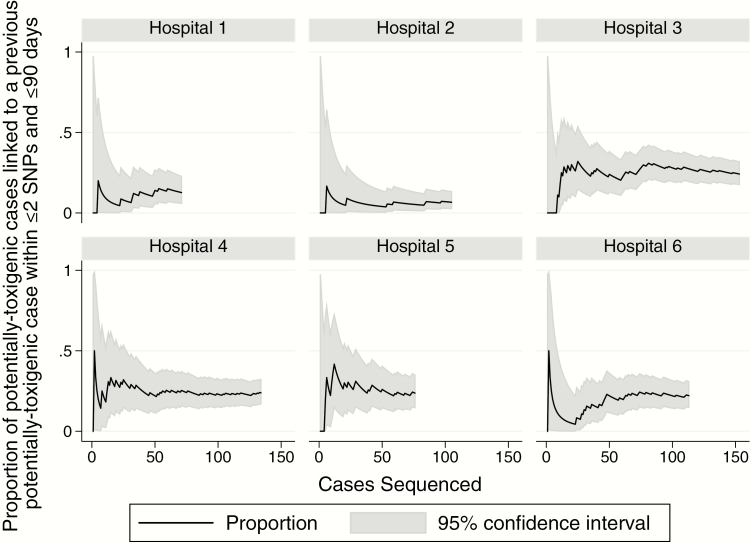
Proportion of potentially toxigenic cases linked to a previous potentially toxigenic case by hospital and number of sequences obtained. Abbreviation: SNP, single-nucleotide polymorphism.

There was no evidence that the number of linked cases varied during the study at any hospital (Figure 1D). Because different numbers of sequences were obtained from the different hospitals, we investigated how this affected the estimated proportions of cases linked to a prior case. Estimated proportions of linked cases were relatively stable once approximately 50 cases had been sequenced (Figure 2).

### Impact of Testing Frequency

**Figure 3.**
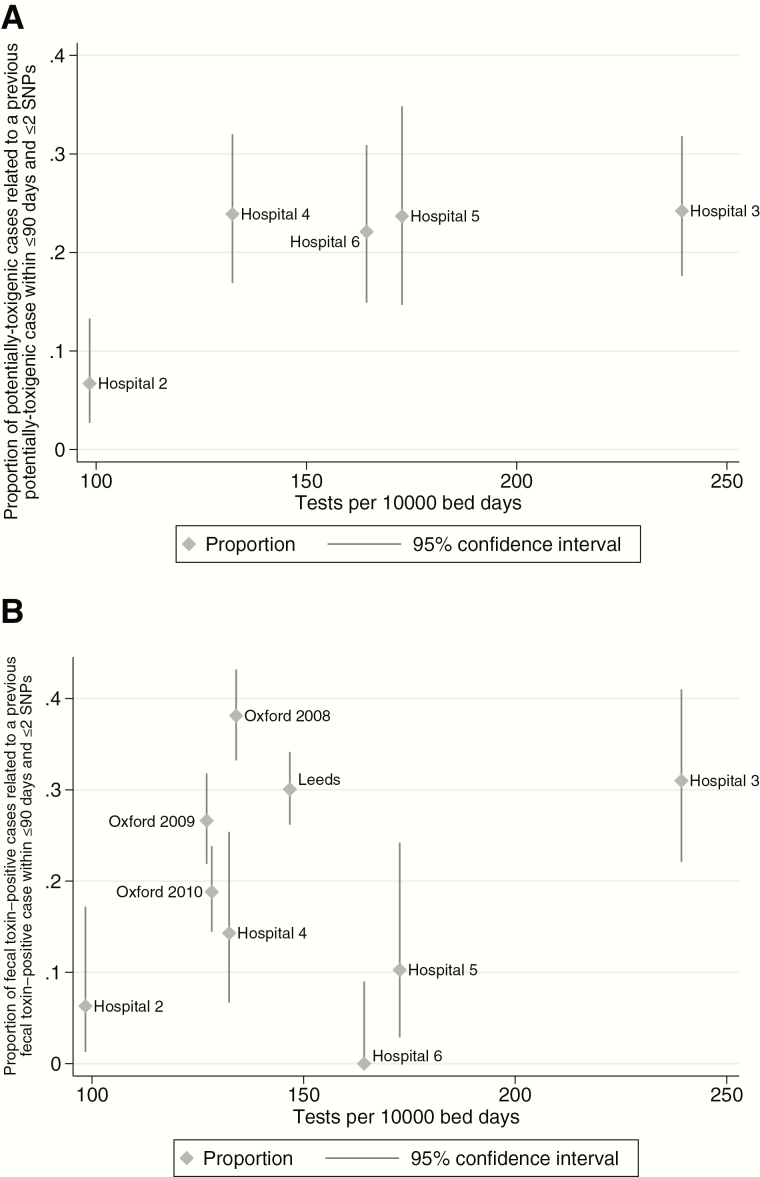
Relationship between the proportion of cases linked to a previous case by *Clostridium difficile* testing rate. *A*, The proportion of potentially toxigenic cases linked to a previous potentially toxigenic case. *B*, The proportion of fecal-toxin-positive cases linked to a previous fecal-toxin-positive case and includes comparisons from previous published data from Oxford (by calendar year) and Leeds (2010–12). Abbreviation: SNP, single-nucleotide polymorphism.

The proportion of originally tested samples that were stored and then culture-positive was similar across the 5 hospitals with testing data, 3.8%–4.3% (*P* = .89, Table 1). In contrast, testing rates ranged from 98 to 239 samples per 10000 bed-days. There was no association between the estimated proportion of cases linked to a previous case within 90 days and testing rates (*P* = .19 for all potentially toxigenic cases, Figure 3A, and P = .60 for fecal-toxin-positive cases only, Figure 3B). For comparison, Figure 3B also displays rates of linked cases for previously published data from Oxford and Leeds.

### Adjustment for Ribotype

**Figure 4.**
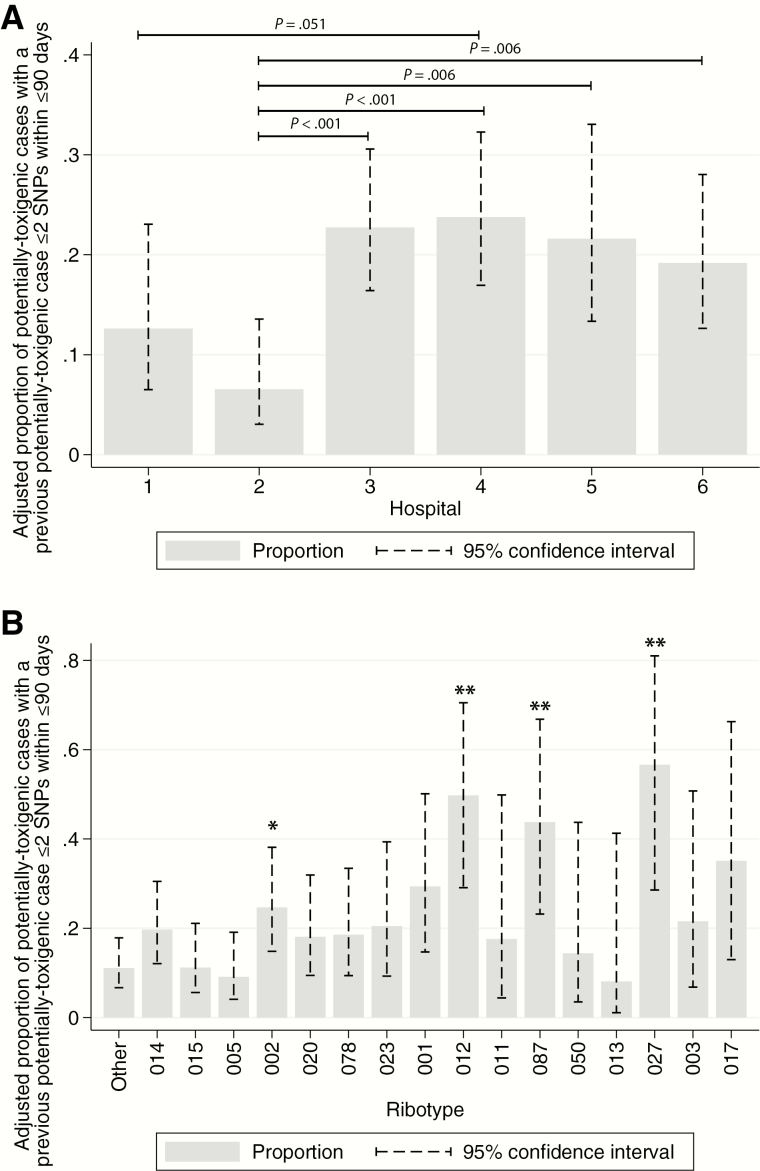
Proportion of cases linked to a previous case by hospital, adjusted for ribotype (*A*), and by ribotype, adjusted for hospital (*B*). In panel *B*, * indicates *P* < .05 compared to the “Other” ribotype category, and ** *P* < .01. Abbreviation: SNP, single-nucleotide polymorphism.

After adjustment for locally circulating ribotypes, estimates of the proportion of potentially toxigenic cases related to a previous potentially toxigenic case within ≤2 SNPs and ≤90 days remained largely unchanged (Figure 4A). Using the same model, per-ribotype estimates for the proportion of related cases, adjusted for differences across hospitals, showed more variation (Figure 4B, Table 2 for unadjusted proportions). Ribotype-027 had significantly more related cases (adjusted proportion, 57%, 95% CI 29–81%, n = 12) than the comparison group of all other ribotypes (11%, 7–18%, *P* = .002, n = 124), as did ribotype-002 (25%, 15–38%, *P* = .04, n = 53), 012 (50%, 29–71%, *P* = .001, n = 22), and 087 (44%, 23–67%, *P* = .005, n = 18).

### Adjustment for Completeness of Testing

As only 144/178 (81%) of GDH-positive samples at hospital 2 were retrievable for culture we assessed the likely impact of these missing samples on the estimated proportion of linked cases by simulating transmission and sampling at a theoretical hospital (Figure S1). As sampling becomes increasingly less complete, the estimated proportion of linked cases declines proportional to the probability of a case being sampled. Applying our simulation to hospital 2 provides a revised estimate of 8% of cases being linked to a prior case (see Supplement for details).

## DISCUSSION

Here, we demonstrate the value of WGS as a tool to estimate different rates of *C. difficile* transmission across institutions. Sequencing consecutive *C. difficile* isolates from routine testing over one year, we found transmission rates varied between 6 hospitals. Considering all patients with potentially toxigenic *C. difficile*, irrespective of fecal-toxin status, in the best performing hospital only 7% of patients’ isolates were sufficiently genetically related to a previous isolate from another patient to support transmission (8% adjusting for incomplete sampling). By contrast, approximately 3–4-fold more isolates (22–26%) were related in 4 of the other hospitals. These results remained similar after adjusting for the locally circulating strains.

Restricting to only patients with fecal-toxin-positive CDI, we confirmed previous findings that only a minority of CDI cases arise from contact with another symptomatic case: 35% in Oxford [10], 35% in Leeds [11], and 37% of ribotype-027 cases in Liverpool [12], were genetically linked to a previous case, with only a subset of these cases sharing time and space on the same hospital ward. Applying the criteria for linking cases used in the present study to the Oxford and Leeds data sets, 38% of cases in Oxford were linked to a previous case in 2008 falling to 19% in 2010, and 30% of cases were similarly linked in Leeds. Across the 6 study hospitals, serving a range of populations, toxin-positive CDI linkage rates were all <15% with the exception of hospital 3, where 31% of cases were linked. It is likely the lower linkage rates in the current study in part reflect the falling incidence of ribotype-027 [11], associated with more onward transmission in this study, likely as a result of national fluoroquinolone restriction [27] but may also represent changes in infection prevention and control practice.

Our findings also support the recently reported role in transmission of GDH-positive patients with toxigenic *C. difficile*, but no detected fecal-toxin [28]. By sequencing all GDH-positive cases, we were able to compare the probability of fecal-toxin-positive and toxin-negative patients being potential sources of transmission, that is, having *C. difficile* genetically linked to a subsequent *C. difficile* isolate in another patient. Fecal-toxin-negative patients were similarly infectious to fecal-toxin-positive patients: fecal-toxin status did not affect the odds of being a potential transmission source. Strategies to identify and institute infection control measures around patients with potentially toxigenic *C. difficile* without detected fecal-toxin are therefore likely to reduce overall CDI incidence, although may be more costly, for example if toxin gene PCR is used as an initial screen rather than GDH EIA. Toxin-positive patients, that is, CDI cases, were more likely to have an identified potential transmission donor, than toxin-negative patients. This is in keeping with previous observations that recent *C. difficile* acquisition is associated with increased risk of disease, whereas long-term carriage is relatively protective [29].

It is likely that differing clinical CDI testing thresholds applied across the study hospitals, despite each being guided by national recommendations; notably, testing rates varied more than 2-fold between hospitals (98–239 tests/10000 bed-days). However, despite this variation, the overall proportion of samples tested that were *C. difficile* culture-positive was very similar across hospitals (~4%). These 2 findings combined resulted in varying rates of potentially toxigenic *C. difficile* isolation, 4.2–8.2/10000 bed-days, and varying (fecal-toxin-positive) CDI rates, 1.8–5.7/10000 bed-days. As the proportion of samples that were *C. difficile* culture-positive was close to reported community asymptomatic *C. difficile* colonization rates (~4%), and lower than reported colonization rates in asymptomatic hospital inpatients, (~10%) [30], it is possible that the higher reported CDI rates in some study hospitals may reflect overascertainment; independent assessment of which symptomatic patients are tested for CDI would be required to resolve this with certainty [7]. As designed, the study did not measure the extent of transmission involving asymptomatic patients, and therefore it is likely that not all hospital-associated transmission is captured. However, as this was the case for all hospitals, comparisons can still be made between hospitals and with previous studies investigating symptomatic patients.

Interestingly, we did not find any evidence of a relationship between rates of *C. difficile* testing and proportions of cases that could be linked to a previous case. Differing sampling/testing will likely mean the study populations at each hospital varied, for example with some institutions potentially more likely to include milder CDI cases than others. It should also be noted that differences in the population sampled by a particular testing strategy may affect the proportion of cases linked differently to incomplete sampling of a given population. We quantified the impact of the latter through simulation. Unfortunately, incomplete sampling could appear very similar to the impact of good infection control, as both results in low proportions of linked cases. One study limitation is that we only sequenced 81% GDH-positive samples at hospital 2. However, we demonstrate it may be possible to adjust for incomplete sampling, providing missed cases as assumed missing at random, and the number of onward transmissions from each case was random.

Both a limitation and a strength of our approach is that it relies only on sequencing laboratory samples and sampling dates. We demonstrate this allows comparative hospital surveillance with very limited, and no personal, sensitive or confidential, data. However, without ward admission and patient contact data, it is possible some genetically linked cases do not represent direct transmission from other cases. Genetic links might also arise through indirect healthcare-associated transmission via unsampled hosts or the hospital environment. Additionally, a minority of cases, without healthcare exposure in the last 90 days, may still have been genetically linked. However, there is no obvious reason why genetically related community *C. difficile* exposures, and therefore the proportion of such cases linked, should vary across England at a population level, even if other CDI risk factors do vary geographically, for example, antimicrobial use. Therefore, although we analyze transmission within the populations served by each hospital, as most CDI cases have recent healthcare exposure, the overall proportion of linked cases is still likely to be a reasonable combined indicator of infection control performance around cases and more generally. Without patient-level identifiers some repeat tests from the same patient may have been wrongly assigned as transmission events; however, we anticipate this was uncommon; repeat testing within 28 days is discouraged in national guidelines [17], and such samples are frequently not routinely processed.

Our method of comparing infection control performance depends on culturing *C. difficile*, which is not routinely undertaken, and on sequencing at least 6 months of samples, at around US$100 per sample. However, if samples are stored, as recommended in England, *C. difficile* could be cultured and sequenced retrospectively if increased incidence was noted and then continued prospectively to monitor the impact of any interventions. The cost-effectiveness of such an approach needs further evaluation.

In summary, here we present a novel method that enables assessment of the extent of hospital-acquired infection transmission within healthcare institutions. This approach revealed differences in CDI transmission rates across 6 English hospitals. It demonstrates the potential of whole-genome sequencing as a nationwide tool to identify institutions with excellent and also suboptimal infection control and therefore has the potential to allow targeted efforts to reduce CDI incidence.

## Supplementary data

Supplementary materials are available at *Clinical Infectious Diseases* online. Consisting of data provided by the authors to benefit the reader, the posted materials are not copyedited and are the sole responsibility of the authors, so questions or comments should be addressed to the corresponding author.

## Supplementary Material

cdlink_supplement_20170313

## Notes

***Acknowledgments.*** Albert Lessing and Shokor Shams for assistance from Heatherwood & Wexham Park Hospitals, Slough, UK.

***Financial support.*** This work was supported by Public Health England and the National Institute for Health Research Oxford Biomedical Research Centre. D. W. C. and T. E. A. P. are National Institute for Health Research senior investigators. D. W. E. is a National Institute for Health Research clinical lecturer.

***Data deposition.*** Sequences generated during this study can be found on the NCBI short read archive under BioProject PRJNA369188.

***Potential conflicts of Interest.*** M. H. W. has received consulting fees from Actelion, Astellas, MedImmune, Merck, Pfizer, Sanofi-Pasteur, Seres, Summit, and Synthetic Biologics; lecture fees from Alere, Astellas, Merck & Pfizer; and grant support from Actelion, Astellas, bioMerieux, Da Volterra, Merck and Summit. S. D. G. has received consulting fees from Abbott, Aquarius Population Health, Astellas and MSD; lecture fees from Astellas, MSD and Orion Diagnostics; and grant support from Astellas. No other author has a conflict of interest to declare. All authors have submitted the ICMJE Form for Disclosure of Potential Conflicts of Interest. Conflicts that the editors consider relevant to the content of the manuscript have been disclosed.
