# Supplementary material for: Comparison of Control of Clostridium difficile Infection in Six English Hospitals Using Whole-Genome Sequencing
Source: Clin Infect Dis. 2017 May 29;65(3):433–41. doi: 10.1093/cid/cix338 (PMC5850028; doi:10.1093/cid/cix338)
Supplement: cdlink_supplement_20170313 [file cix338_suppl_cdlink_supplement_20170313.docx]

# Comparison of control of *Clostridium difficile* infection in six English hospitals using whole-genome sequencing: Supplementary Materials

## Methods

### Samples and settings

Six hospital laboratories were studied, including a tertiary referral center and teaching hospital, Guy’s & St Thomas’ NHS Foundation Trust, London, and five district general hospitals serving a mix of urban and rural populations: Great Western Hospitals NHS Foundation Trust, Swindon, Wiltshire; Heatherwood and Wexham Park Hospitals NHS Foundation Trust, Berkshire; St Helens & Knowsley Hospitals NHS Trust, Merseyside; Calderdale & Huddersfield NHS Foundation Trust, Yorkshire; and City Hospitals Sunderland NHS Foundation Trust, Sunderland.

Each hospital used a combination of tests in line with the UK recommended *C. difficile* testing guidance. Hospital-1 used toxin gene PCR as a screening test (BD MAX C difficile PCR, BD, Franklin Lakes, NJ, USA), followed by confirmatory fecal toxin enzyme-linked fluorescent assay (ELFA) (bioMérieux Vidas C. difficile toxin AB, bioMérieux UK, Basingstoke, UK). Hospital-2 used a combined enzyme immunoassay (EIA) to detect glutamate dehydrogenase (GDH) and fecal toxin (TechLab C diff Quik Check Complete, Techlab, Blacksburg, VA, USA). Hospital-3 used a combined EIA to detect GDH and fecal toxin (TechLab C diff Quik Check Complete, Techlab, Blacksburg, VA, USA). Hospital-4 used separate GDH and fecal toxin EIA tests (TechLab C diff Chek-60 and TechLab C diff Tox A/B II, respectively, both Techlab, Blacksburg, VA, USA). Hospital-5 used a GDH EIA (Prolab GDH EIA, Pro-Lab Diagnostics, Toronto, Canada), a fecal toxin ELFA (bioMérieux Vidas C. difficile toxin AB, bioMérieux UK, Basingstoke, UK). Hospital-6 used separate GDH and fecal toxin EIA tests (TechLab C diff Quik Chek, and TechLab C diff Quik Chek TOX AB respectively, both Techlab, Blacksburg, VA, USA). Toxin gene PCR (Cepheid GeneXpert, Cepheid, Sunnyvale, CA, USA) was also performed on all samples at hospital-2, -3, and -6, and on samples from inpatients at hospital-5.

### Sequencing

Sequences were compared using single nucleotide polymorphisms, SNPs, obtaining differences between sequences from maximum likelihood phylogenies constructed using PhyML[1] (with generalized time-reversible substitution model and “BEST” tree topology search algorithm), corrected for the effect of recombination using ClonalFrameML[2] (with default settings). Sequence reads were also assembled *de novo* with Velvet*,*[3] and toxigenic strains identified using BLAST searches of *de novo* assemblies (≥1000 nucleotide identities with toxin A or B genes).

### Risk factors

To assess whether the locally circulating strain mix affected estimates of the proportion of cases linked to a previous case, hospital-specific estimates were adjusted for ribotype using multivariate logistic regression. For each case occurring after the first 90 days at each hospital, the modelled outcome was genetic linkage with any previous case within ≤2 SNPs and ≤90 days. Ribotypes with 15 or more observations were considered as separate categories; ribotypes with less than 15 observations were combined into a single ‘other’ category, used as the reference. Marginal mean estimates for the proportion of cases related to a previous case either by hospital or by ribotype were obtained averaging over the distribution of the other factor (Stata “margins” command). All analyses were performed using Stata 14.1 (Stata Corp, College Station, TX).

### Simulations

To assess the impact of incomplete sampling we simulated CDI cases occurring in patients at a hospital. Each simulation was run for 500 days, with 500 “introductions” (cases not related to a prior case through direct transmission) of CDI to the hospital randomly distributed throughout the time period. Simulating forward in time, at daily intervals, on each day, each newly diagnosed case had a random chance of resulting in a secondary case according to a specified probability. Where a secondary case occurred, the time delay between the diagnosis of the first and secondary cases was sampled from a gamma distribution (parameters shape=12, scale=2, equivalent to a median [IQR] 23 [19-28] days between transmitted cases; estimates were insensitive to the choice of parameter values provided the median generation interval remained less than 90 days). Each secondary case could then in turn potentially result in further secondary cases. Having run each simulation, a proportion of cases were randomly removed from the dataset once. Only secondary cases where the transmission source remained in the dataset were considered linked to a prior case and only cases from 90 days into the simulation were evaluated for a possible source. Using this approach, we assessed the impact of sampling 100%, 90%, 80%, 70%, 60%, and 50% of true cases, on the proportion of observed cases linked to a prior observed case, when the underlying probability of a secondary case was 0.25 and 0.5. Each simulation was run 1000 times and performed in R version 3.2.1.

## Results

### Simulations

As only 144/178 (81%) of GDH-positive samples at hospital-2 were retrievable for culture we assessed the likely impact of these missing samples on the estimated proportion of linked cases by simulating transmission and sampling at a theoretical hospital (Figure S1). As sampling becomes increasingly less complete, the estimated proportion of linked cases declines proportional to the probability of a case being sampled. This potentially allows for correction of incomplete sampling if the observed proportion of linked cases is multiplied by 1/(completeness of testing) to provide an adjusted number of linked cases. Applying this hospital-2 provides a revised estimate of 8% of cases being linked to a prior case; still considerably lower than the majority of the other hospitals.

**Figure S1. Simulated number of cases linked to a previous case according to completeness of sampling.** The median from 1000 simulations is plotted, the dashed bars show the 2.5^th^ to the 97.5% percentiles.

## References

1. Guindon S, Gascuel O. A simple, fast, and accurate algorithm to estimate large phylogenies by maximum likelihood. Syst Biol **2003**; 52:696–704.

2. Didelot X, Wilson DJ. ClonalFrameML: efficient inference of recombination in whole bacterial genomes. PLoS Comput. Biol. **2015**; 11:e1004041.

3. Zerbino DR, Birney E. Velvet: Algorithms for de novo short read assembly using de Bruijn graphs. Genome Res **2008**; 18:821–829.
